# Air gun wound: bihemispheric penetrating brain injury in a paediatric patient

**DOI:** 10.1259/bjrcr.20180070

**Published:** 2018-11-14

**Authors:** Andre Tjie Wijaya, I Made Dwijaputra Ayusta, I Wayan Niryana

**Affiliations:** 1 Department of Radiology, Faculty of Medicine Udayana University/Sanglah General Hospital, Denpasar, Indonesia; 2 Department of Surgery, Neurosurgery Division, Faculty of Medicine Udayana University/Sanglah General Hospital, Denpasar, Indonesia

## Abstract

Air guns are classified as low-velocity missiles and they usually considered safe and harmless. Despite that fact, air guns still can make serious or life-threatening injuries. Most of air gun injuries occur in paediatric population. A 2-year-old boy was shot in the forehead withan air gun accidentally. Skull radiography and non-contrast CT scan of the head were performed and showed penetrating bihemispheric brain injury from the left frontal to right occipital lobes at the level of the lateral ventricle with a metal-density foreign body at the right occipital. A projectile was successfully extracted via craniotomy, without complications. Air guns have the potential to cause fatal, life-threatening injury especially in children. Imaging is crucial for the evaluation of wound ballistics. Understanding about the mechanism of projectiles and wound ballistics is very helpful for radiologists to conceptualize these injuries when interpreting these cases. The role of radiology in ballistic wound cases is critical and important, both for clinical and forensic settings.

## Clinical presentation

A 2-year-old boy was shot in the forehead with an air gun accidentally. Unfortunately, the parents did not know what kind of air gun was used because it was not theirs. The patient was fully alert and complained about headaches. There was no history of decrease of consciousness, seizure, or extremity weakness after the incident. Physical examination revealed normal vital signs, no signs of neurological deficit, and active bleeding from the wound at the left frontal cortex. The patient was evacuated to Ruteng District General Hospital, Nusa Tenggara Timur, Indonesia. Then the patient was referred to Sanglah General Hospital, Bali, for further management. It took approximately 2 h and 50 min by airplane from Ruteng to Denpasar. The patient came to our centre 2 days after the incident.

## Investigations

**Figure 1.**
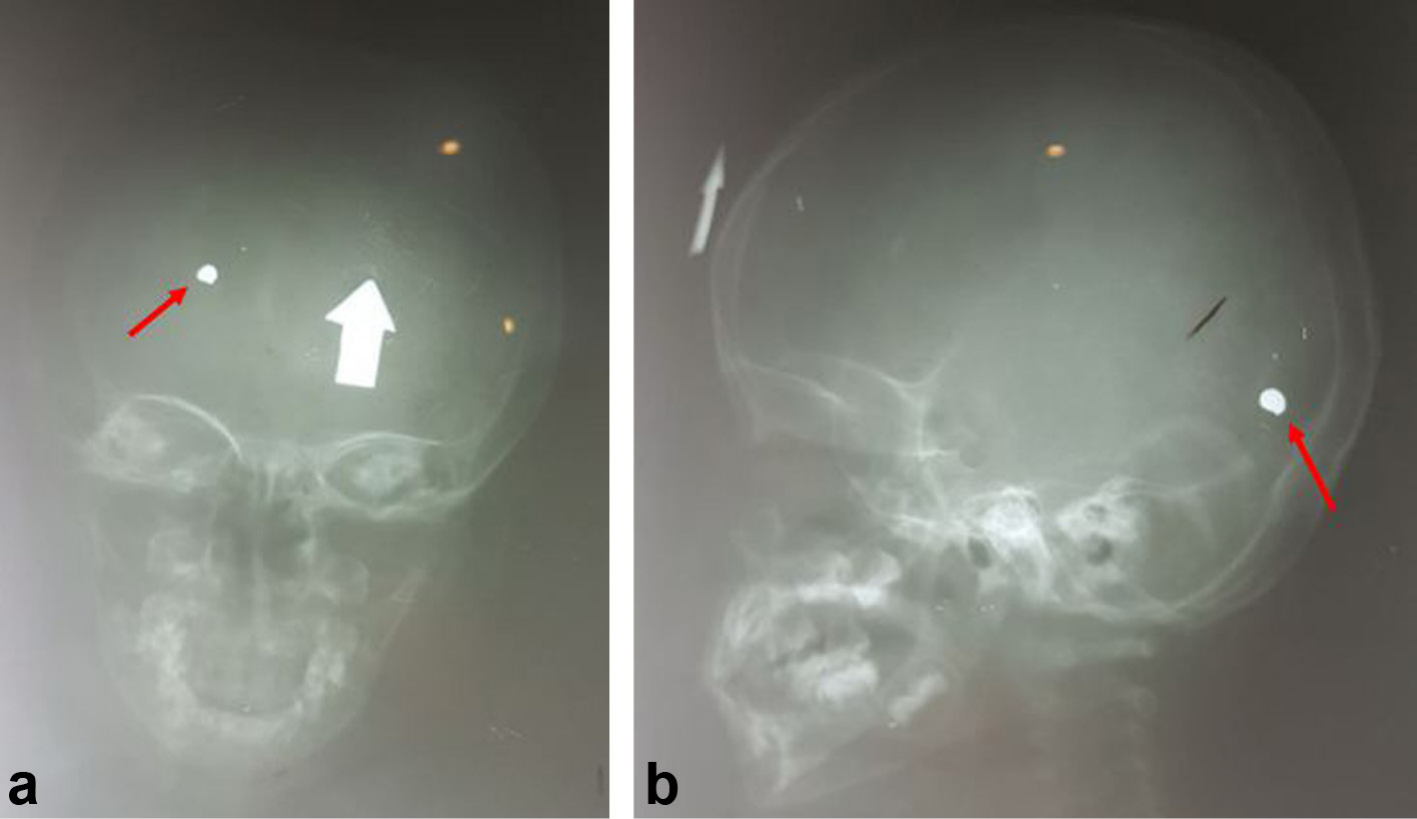
Skull radiograph anteroposterior/lateral (a, b). A metal-opacity foreign body (red arrow) is detected at the right occipital lobe with fracture at the left frontal bone. A marker must be used in radiography to mark the entry wound (white arrow).

**Figure 2.**
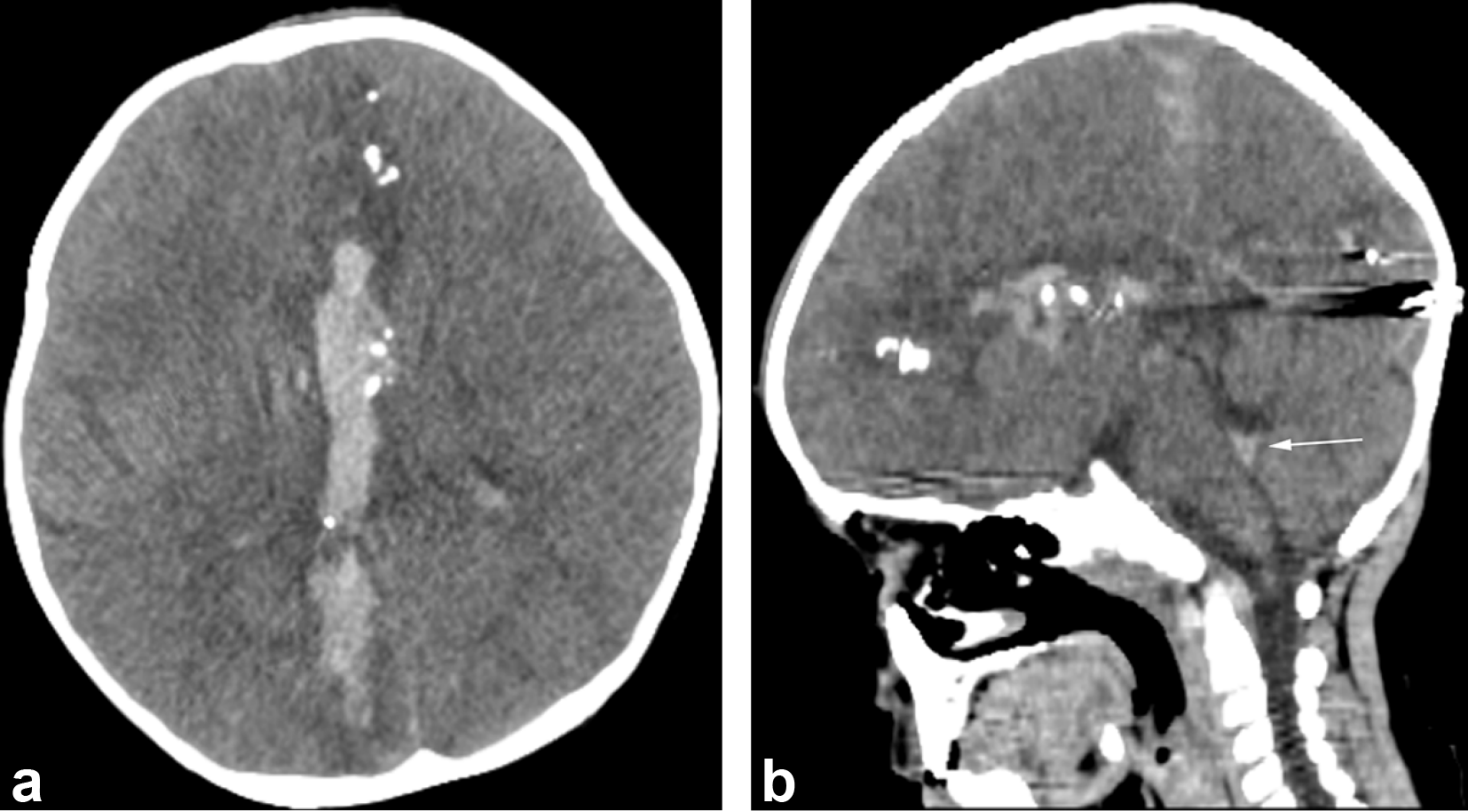
Initial non-contrast CT scan of the head. (a) The axial section shows an intracranial hemorrhage from the left frontal to right occipital lobe with bone and metal fragments found along the path, and metal-density (HU 4000) at the right occipital region. (b) The sagittal section shows that the projectile path occurred at the level of the lateral ventricle, with a foreign body at the occipital region. Note that there is blood in the fourth ventricle as well (arrow). HU, Hounsfield unit.

**Figure 3.**
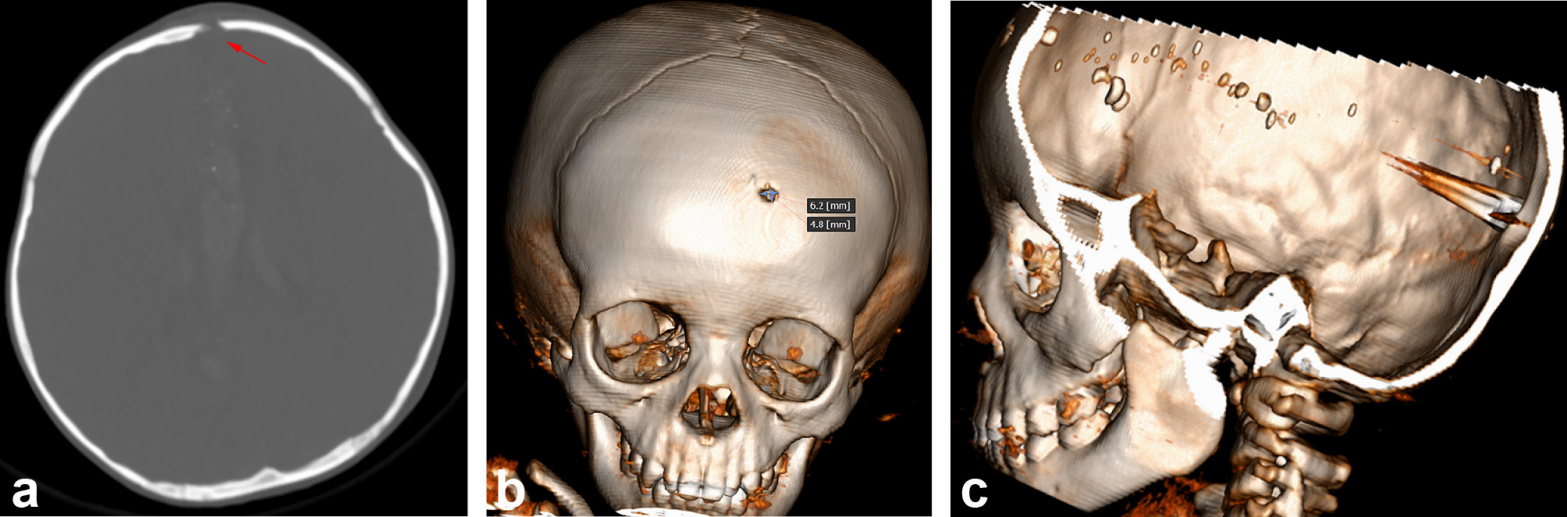
Bone window and VRT. (a) The axial section of the bone window shows a bone fracture at the left frontal bone. Note there is a bevel at the internal table (internal or inward beveling), which is consistent with the characteristics of an entry site (red arrow). (b) An anterior view of the VRT reveals a bone fracture (6.2 × 4.8 mm). (c) The image shows a metal-density foreign body crossing from the entry site at the left frontal to right occipital lobes, with a metal artefact and fragments along the projectile tract. VRT, volume rendering technique.

Skull radiography showed a metal-opacity foreign body at the right occipital region (Figure 1). A non-contrast CT (NCCT) scan of the head was performed and revealed a projectile path from the left frontal to right occipital lobes at the level of the lateral ventricle with a metal-density foreign body at the end of the path, as well as intracranial haemorrhage with bone and projectile fragments along that path. Intraventricular haemorrhage was evident in ventricles I–IV and cerebral oedema was also present. Fracture with internal beveling was detected at the left frontal bone (Figures 2 and 3). There was no sign of brain herniation. Projectile extraction was scheduled 2 weeks later at a central operating theater with C-arm guiding.

2 weeks later, before the surgery, a follow-up head NCCT was performed. There was a late, subacute phase intracranial haemorrhage from the left frontal to right occipital region. Migration of projectile fragments were not detected. During hospitalization, there were no signs of deterioration. His vital signs were within normal limit, fully conscious (GCS 15), and no neurological defects or pathological reflexes were occurred.

## Treatment

**Figure 4.**
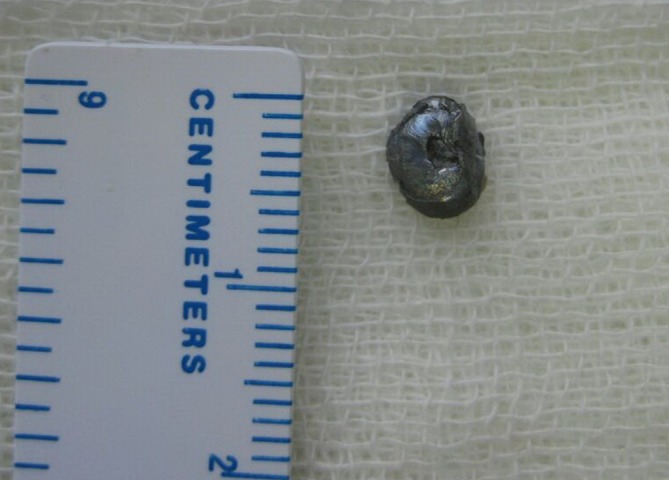
Deformed projectile (diameter of 6 mm) was successfully extracted with C-arm guided craniotomy.

Local wound care was performed at the entry site. Then the patient was treated with analgesic (paracetamol) and antibiotic (ceftriaxone). C-arm-guided craniotomy surgery was done and a deformed-projectile was successfully extracted from the right occipital region (Figure 4) . On the seventh day, the patient was discharged without any complications. No rehabilitation processes were done. There was also no sign of infection or neurological impairment.

## Discussion

Our case is an accidental case about head air gun wound in a child. The case is a reminder for us to prevent the same accident in future. Imaging plays an essential role in ballistic wound cases, for clinical and medico-legal settings.

Airguns are categorized as low-velocity missiles (muzzle velocity <300 m/s), but even 60–100 m/s is enough to fracture bone.^1–3^ About 80–90% of air gun injuries occur in population with age under 19 years, especially boys, with most of them are caused by the gun’s careless use.^4,5^ Air guns, that are considered toys or sport equipments, have the potential to cause fatal, life-threatening injury. The case reported here is one example of an accidental air gun injury in a boy who plays air gun without supervision. In this case, the projectile penetrated the skull at frontal bone without exiting the skull or called penetrating wound.

The trajectory of the projectile in this case was from left frontal to right occipital region. It supported with fracture at left frontal bone with internal beveling and the projectile in right occipital region. Inward or internal beveling indicates the point of entry, and exit site is marked by an outward or external bevel.^6–10^ Determine the trajectory of the projectile is important to predict tissue damages and in medicolegal issues. In some cases, determination of the bullet’s path is difficult, *e.g.* gunshot in the abdomen or if the bullet has ricochet. If there is a suspicion of abnormal trajectory, ordering multiple conventional radiographs at different parts of body or a full body CT can be helpful.^11^ It takes experience and ballistic knowledge to make a correct radiology reports about gunshot cases. An incorrect one can be a serious problem in medicolegal setting. It is better to keep the report as simple as can be if radiologists not sure about projectile trajectory or projectile type.

Skull X-ray and head NCCT were performed on the patient. We placed a marker on the entry wound (left frontal area), in accordance with the previous published work.^7^ A CT scan is considered the gold-standard; it is very informative given its capabilities to accurately determine the delineation of the projectile tract, while also evaluating the type and extent of visceral injuries.^12,13^ Radiologists should evaluate migration of projectiles if follow-up examinations are performed. One disadvantage of CT in ballistic wound cases is the presence of beam hardening artifacts, but can be reduced with dual energy CT. Unfortunately, CT scan in our institution (Sanglah General Hospital, Denpasar, Bali, Indonesia) cannot perform dual energy examination. Without dual energy CT, we can still evaluate intracranial and bone damage, path of the projectile, and signs of migration on the follow-up NCCT.

MRI was not performed because of the risk of secondary dislocation. Theoretically, MRI is only used if the ferromagnetic features of the projectiles can be confirmed without question.^7^ But, even for experts it can be difficult to determine what kind of ammunition was used. Due to the risk of secondary dislocation, the usage of MRI should not be advised in patients with metallic foreign bodies or gunshot bullets.

There are limited clear indications to perform removal of all bullet fragments. Clear indications for bullet removal, are fragment movement, abscess formation, vascular compression, and hydrocephalus.^14^ Because of lacking of facility, the surgery was not performed at the first centre. When the patient came to our centre 2 days after the incident, there were no signs of neurological impairment and infections. We decided to observe the patient closely and treat conservatively. Management of intracranial gunshot in paediatric population relying on guidance from the adult literature because of shortage of relevant research.^15^ Emergency surgery was indicated for evacuation of a spacious intracranial haemorrhage with midline shift (>5 mm), elevation of intracranial pressure, and to manage infection if there was noticeable herniation of brain from the projectile wound.^16,17^ None of those indications were found on our patient.

## Learning points

1. Numerous imaging modalities can be used to evaluate air gun injury, but CT scan is considered as the goldstandard. MRI should not be advised in such cases.
2. The patient’s clinical presentations were within normal limit even there was a terrible-look brain injury because of their neurological plasticity. Compare to adults, children have greater neurological restoration and the mortality rate is also lower.
3. Although air guns are considered toys or sporting equipment, they still possess a potential for danger. Most cases in children happen accidentally. Our awareness should be increased to prevent accidents such as those reported in the present case study.
